# Association of Arterial Stiffness and Electrocardiography-Determined Left Ventricular Hypertrophy with Left Ventricular Diastolic Dysfunction

**DOI:** 10.1371/journal.pone.0049100

**Published:** 2012-11-07

**Authors:** Po-Chao Hsu, Wei-Chung Tsai, Tsung-Hsien Lin, Ho-Ming Su, Wen-Chol Voon, Wen-Ter Lai, Sheng-Hsiung Sheu

**Affiliations:** 1 Division of Cardiology, Department of Internal Medicine, Kaohsiung Medical University Hospital, Kaohsiung Medical University, Kaohsiung, Taiwan; 2 Faculty of Medicine, College of Medicine, Kaohsiung Medical University, Kaohsiung, Taiwan; 3 Department of Internal Medicine, Kaohsiung Municipal Hsiao-Kang Hospital, Kaohsiung Medical University, Kaohsiung, Taiwan; University of Illinois at Chicago, United States of America

## Abstract

**Objectives:**

Increased arterial stiffness is associated with left ventricular diastolic dysfunction (LVDD), but this association may be influenced by left ventricular (LV) performance. Left ventricular hypertrophy (LVH) is not only a significant determinant of LV performance, but is also correlated with LVDD. This study is designed to compare LV diastolic function among patients divided by brachial-ankle pulse wave velocity (baPWV) and electrocardiography (ECG)-determined LVH and to assess whether increased baPWV and ECG-determined LVH are independently associated with LVDD.

**Methods:**

This cross-sectional study enrolled 270 patients and classified them into four groups according to the median value of baPWV and with/without ECG-determined LVH. The baPWV was measured using an ABI-form device. ECG-determined LVH was defined by Sokolow-Lyon criterion. LVDD was defined as impaired relaxation, pseudonormal, and restrictive mitral inflow patterns. Groups 1, 2, 3, and 4 were patients with lower baPWV and without ECG-determined LVH, lower baPWV but with ECG-determined LVH, higher baPWV but without ECG-determined LVH, and higher baPWV and with ECG-determined LVH respectively.

**Results:**

Early diastolic mitral velocity (Ea) was gradually decreased from group 1 to group 4 (p≦0.027). Patients in group 4 had the highest prevalence of LVDD (all p<0.001). After multivariate analysis, both baPWV and ECG-determined LVH were independent determinants of Ea (β = −0.02, P<0.001; β = −1.77, P<0.001 respectively) and LVDD (odds ratio = 1.02, P = 0.011 and odds ratio = 3.53, P = 0.013 respectively).

**Conclusion:**

Our study showed the group with higher baPWV and ECG-determined LVH had the lowest Ea and highest prevalence of LVDD. In addition, both baPWV and ECG-determined LVH were independently associated with Ea and LVDD. Hence, assessment of arterial stiffness by baPWV and LVH by ECG may be useful in identifying the high risk group of LVDD.

## Introduction

Increased pulse wave velocity (PWV), which reflects increased arterial stiffness, may result in left ventricular (LV) remodeling and thereby cause left ventricular diastolic dysfunction (LVDD) [1]–[2]. Masugata et al. found that brachial-ankle PWV (baPWV) was significantly correlated with LVDD [3]. Abhayaratna et al. [4] demonstrated that aortic PWV progressively increased according to the severity of LVDD. Several previous studies have also reported a relation between arterial stiffness and LV diastolic function [5]–[7]. However, a reducing LV ejection velocity may decrease the PWV. Weber et al. [8] and our recent studies [9]–[10] found LV performance could influence the PWV. Left ventricular hypertrophy (LVH) is a significant determinant of impaired LV performance [11]. Therefore, LVH may have a significant influence on the relationship between PWV and LV diastolic function. The 12-lead electrocardiography (ECG) is a simple and inexpensive noninvasive clinical tool to assess LVH. LVH is reported to be correlated with LVDD [12]–[14]. In patients with LVH caused by pressure overload, elevated LV end-diastolic pressure relative to a normal or small LV diastolic cavity volume is a hemodynamic hallmark and advanced hypertrophy frequently causes a pseudonormal/restrictive pattern LVDD [15]. Villari BM et al. stated that the prolongation of relaxation was closely related to the magnitude of hypertrophy in patients with aortic stenosis and regurgitation [12]. Hess OM et al. also reported that the most common cause for LVDD is LVH in patients with aortic stenosis [13]. These studies all revealed that there was a close association between LVH and LVDD.

A clinical device, ABI-form (Colin VP1000, Komaki, Japan), has been developed to automatically and simultaneously measure blood pressure (BP) in both arms and ankles and record pulse waves of the brachial and posterior tibial arteries using an automated oscillometric method. Using this device, we can easily obtain baPWV values [16]. Accordingly, the aims of this study are to compare the LV diastolic function among patients divided by the median value of baPWV and with/without ECG-determined LVH and to assess whether the increased baPWV and 12-lead ECG-determined LVH are independently associated with LVDD.

## Materials and Methods

### Study Subjects

This was a cross-sectional study. Study subjects were randomly included from a group of patients who arranged for echocardiographic examinations at Kaohsiung Municipal Hsiao-Kang Hospital. Patients with significant aortic or mitral valve disease, atrial fibrillation, or inadequate image visualization were excluded. We did not consecutively include all the patients because 12-lead ECG and baPWV measurement must be performed within 10 minutes after the completion of echocardiographic examination. Finally, 270 consecutive patients who agreed to have immediate ECG and baPWV were included in this study. The study population was further classified into 4 groups on the basis of the median value of baPWV and with/without ECG-determined LVH. Groups 1, 2, 3, and 4 were made up of patients with lower baPWV and without ECG-determined LVH, with lower baPWV but with ECG-determined LVH, with higher baPWV but without ECG-determined LVH, and with higher baPWV and with ECG-determined LVH respectively. All patients were in sinus rhythm.

### Ethics Statement

The study protocol was approved by the Institutional Review Board of the Kaohsiung Medical University Hospital (KMUH-IRB-20120132). Informed consents were obtained in written form from patients and all clinical investigations were conducted according to the principles expressed in the Declaration of Helsinki. The patients gave consent for the publication of the clinical details.

### Collection of Demographic, Medical, and Laboratory Data

Demographic and medical data including age, gender, history of diabetes mellitus, hypertension, and coronary artery disease, body mass index (BMI), systolic BP, diastolic BP, pulse pressure, and heart rate were obtained from medical records or interviews with patients. BMI was calculated as the ratio of weight in kilograms divided by the square of height in meters. Laboratory data including fasting glucose, triglyceride, total cholesterol, and hematocrit were also collected. In addition, information regarding patient medications including angiotensin converting enzyme inhibitors (ACEIs), angiotensin II receptor blockers (ARBs), β-blockers, calcium channel blockers (CCBs), diuretics, and nitrates during the study period was obtained from medical records.

### Echocardiographic Assessment

The echocardiographic examinations were performed by one experienced sonographer using transthoracic echocardiography (Vivid 7, General Electric Medical Systems, Horten, Norway), with the participant respirating quietly in the left decubitus position. Two-dimensional and two-dimensionally guided M-mode images were recorded from the standardized views. The Doppler sample volume was placed at the tips of the mitral leaflets to obtain the LV inflow waveforms from the apical 4-chamber view. All sample volumes were positioned with ultrasonic beam alignment to flow. Pulsed tissue Doppler imaging was obtained with the sample volume placed at the lateral corner of the mitral annulus from the apical 4-chamber view. The wall filter settings were adjusted to exclude high-frequency signals and the gain was minimized. A normal mitral inflow pattern was recognized if the ratio of transmitral E wave velocity (E) to transmitral A wave velocity (A) was ≧ 0.75, early diastolic mitral velocity (Ea) ≧ 8 cm/s, and E/Ea≤10, impaired relaxation mitral inflow pattern if the E/A ratio was <0.75, and pseudonormal/restrictive mitral inflow pattern if the E/A ratio was ≧ 0.75, Ea <8 cm/s or E/Ea >10 [17]–[18]. In this study, LVDD was defined as impaired relaxation and pseudonormal/restrictive mitral inflow patterns. The LV ejection fraction was measured by the modified Simpson’s method. LV mass was calculated using the Devereux-modified method [19]. LV mass index was calculated by dividing LV mass by body surface area. Echocardiographic LVH was defined as suggested by the American Society of Echocardiography/European Society of Echocardiography chamber quantification guidelines [20]. It was defined as left ventricular mass index more than 115 g/m^2^ in men and more than 95 g/m^2^ in women. The raw ultrasound data were recorded and analyzed offline, using software (EchoPAC, GE Medical Systems), by a cardiologist who was blinded to the other data.

### Assessment of baPWV

Within 10 minutes after the completion of the echocardiographic examination, baPWV was assessed using an ABI-form device, which automatically and simultaneously measures BP in both arms and ankles using an oscillometric method [21]–[22]. For measuring baPWV, pulse waves that were obtained from the brachial and tibial arteries were recorded simultaneously and the transmission time, which was defined as the time interval between the initial increase in brachial and tibial waveforms, was determined. The transmission distance from the arm to each ankle was calculated according to body height. The value of baPWV was automatically computed as the transmission distance divided by the transmission time. After obtaining bilateral baPWV values, the average of two values was used for analysis. Systolic and diastolic BPs were measured by the same device. The averages of systolic and diastolic BPs of bilateral arms were used for analysis.

### Assessment of ECG-determined LVH

A standard 12-lead ECG was recorded during quiet respiration, with subjects in a supine position. The ECG was recorded at 25 mm/s and 0.1 mV/mm standardization. The Sokolow-Lyon voltage criterion was used for diagnosis of ECG-determined LVH. It is defined as (SV1+ RV5 or RV6) ≧ 35 mm [23]. Two independent technicians measured the voltage and discrepancies of 2 mm were resolved by a third reader.

### Statistical Analysis

All data were expressed as means (± standard deviation). SPSS 18.0 software (SPSS, Inc., Chicago, Illinois, USA) was used for statistical analysis. Multiple comparisons between study groups were performed by one-way analysis of variance (ANOVA) followed by a post hoc test adjusted with a Bonferroni correction. Categorical and continuous variables between groups were compared by Chi-square analysis and independent-samples *t*-test respectively. The relationship between two continuous variables was assessed by a bivariate correlation method (Pearson’s correlation). Subsequently, significantly correlated variables in the univariate analysis were further analyzed by multiple linear and logistic regression analyses to identify the factors associated with Ea and LVDD respectively. All tests were 2-sided and the level of significance was established as p<0.05.

## Results

The comparison of clinical characteristics and arterial stiffness among the study groups is shown in Table 1. There were 106, 29, 93, and 42 patients in groups 1, 2, 3, and 4, respectively. The median value of baPWV was 1706 cm/s. There were significant differences among the 4 groups in age, gender, prevalence of diabetes and hypertension, heart rate, systolic and diastolic BPs, pulse pressure, and baPWV. The study was performed with patients taking their usual medications. Use of ARBs, CCBs, diuretics, and nitrates were also significantly different among the 4 groups. In addition, there were significant differences among the 4 groups in the laboratory data such as fasting glucose, total cholesterol, and hematocrit.

**Table 1:** Comparison of clinical characteristics and arterial stiffness among study groups.

	Group 1	Group 2	Group 3	Group 4	P value
Number	106	29	93	42	
Age (years)	50±11	48±15	64±12* ^#^	63±12* ^#^	<0.001
Gender (M/F)	55/51	23/6*	44/49#	30/12* ^+^	0.003
DM (%)	15 (14.2%)	8 (27.6%)	35 (37.6%)*	12 (28.6%)*	0.002
HTN (%)	43 (40.6%)	15(51.7%)	69 (74.2%)* ^#^	37(88.1%)* ^#^	<0.001
CAD (%)	10 (9.8%)	6 (20.7%)	21 (23.3%)	8 (20.0%)	0.08
BMI (kg/m^2^)	25.5±3.9	25.9±4.9	25.7±3.4	24.2±3.9	0.175
SBP (mmHg)	126±16	131±18	145±19* ^#^	157±24* ^#+^	<0.001
DBP (mmHg)	76±11	80±12	82±11*	90±12* ^#+^	<0.001
PP (mmHg)	50±12	52±13	63±14* ^#^	67±17* ^#^	<0.001
HR (beat/min)	68±11	76±16*	72±13	74±15*	0.009
baPWV (cm/s)	1392±135	1407±141	1970±301* ^#^	2122±567* ^#+^	<0.001
**Medications**					
ACEIs	17 (16.0%)	7 (24.1%)	15 (16.1%)	10 (23.8%)	0.536
ARBs	19 (17.9%)	9 (31.0%)	40 (43.0%)*	17 (40.5%)*	0.001
β blockers	45 (42.5%)	12 (41.4%)	43 (46.2%)	14 (33.3%)	0.575
CCBs	13 (12.3%)	4 (13.8%)	32 (34.4%)* ^#^	16 (38.1%)* ^#^	<0.001
Diuretics	16 (15.5%)	11 (37.9%)*	15 (16.5%)#	7 (17.5%)#	0.044
Nitrates	25 (24.5%)	11 (37.9%)	34 (37.4%)*	21 (52.5%)*	0.014
**Laboratory parameters**					
Fasting glucose (mg/dL)	105.3±33.8	114.0±35.1	126.8±55.0*	123.6±38.0	0.016
Triglyceride (mg/dL)	151±107	172±114	160±92	142±83	0.679
Total cholesterol (mg/dL)	198.7±47.6	228.3±95.9	193.7±42.9#	189.0±32.7#	0.027
Hematocrit (%)	41.8±5.7	44.1±5.6	40.7±5.3	39.3±6.2#	0.013

ACEIs: angiotensin-converting enzyme inhibitors; ARBs: angiotensin II receptor antagonists; BMI: body mass index; baPWV: brachial-ankle pulse wave velocity; CAD: coronary artery disease; CCBs: calcium channel blockers; DBP: diastolic blood pressure; DM: diabetes mellitus; F: female; HTN: hypertension; HR: heart rate; M: male; PP: pulse pressure; SBP: systolic blood pressure.

* P<0.05 compared with group 1;

# P<0.05 compared with group 2;

+ P<0.05 compared with group 3.

The comparison of echocardiographic characteristics among the study groups is shown in Table 2. The Ea in groups 1, 2, 3, and 4 was 11.1±3.5, 9.4±4.6, 7.9±2.7, and 5.8±2.4 cm/s, respectively. Additionally, the prevalence of LVDD in groups 1, 2, 3, and 4 was 34.9%, 55.2%, 71.0%, and 95.2%, respectively. There were significant differences among the 4 groups in the echocardiographic data such as LV end-diastolic dimension, LV end-systolic dimension, LV ejection fraction, LV mass index, E, A, E/A, E-wave deceleration time, Ea, and E/Ea. Furthermore, Ea was gradually decreased from group 1 to group 4 (p≤0.027). The percentage of LVDD was higher in groups 3 and 4 than in group 1 (both p<0.001) and was higher in group 4 than in groups 2 and 3 (p<0.001 and p = 0.001, respectively).

**Table 2:** Comparison of echocardiographic characteristics among study groups.

	Group 1	Group 2	Group 3	Group 4	P value
LVEDD (mm)	52±8	59±12*	52±8#	57±10* ^+^	<0.001
LVESD (mm)	34±11	43±15*	34±11#	41±12* ^+^	<0.001
EF (%)	61±16	47±19*	58±18#	48±15* ^+^	<0.001
LVMI (g/m^2^)	94±29	137±44*	108±27* ^#^	137±42* ^+^	<0.001
E (cm/s)	84±19	90±25	77±24* ^#^	80±31	0.039
A (cm/s)	70±21	74±24	83±19* ^#^	85±20* ^#^	<0.001
E/A	1.36±0.74	1.36±0.67	1. 20±0.58* ^#^	1.02±0.57* ^#^	0.001
EDT (ms)	178±49	162±64	199±58* ^#^	196±75#	0.006
Ea (cm/s)	11.1±3.5	9.4±4.6*	7.9±2.7* ^#^	5.8±2.4* ^#+^	<0.001
E/Ea	8.6±4.9	11.6±5.8*	11.0±5.6*	15.9±8.7* ^#+^	<0.001
Aa (cm/s)	9.2±3.0	8.1±2.7	9.8±3.5	9.1±3.1	0.089
LVDD	37 (34.9%)	16 (55.2%)	66 (71.0%)*	40 (95.2%)* ^#+^	<0.001
Impaired relaxation mitral inflow pattern	12(11.3%)	3(10.3%)	38(40.9%)* ^#^	17(40.5%)* ^#^	<0.001
Pseudonormal/restrictive mitral inflow pattern	25(23.6%)	13(44.8%)*	28(30.1%)	23(54.8%)* ^+^	0.001

A: transmitral A wave velocity; Aa: late diastolic mitral velocity; E: transmitral E wave velocity; Ea: early diastolic mitral velocity; EDT: E-wave deceleration time; EF: ejection fraction; LVDD: left ventricular diastolic dysfunction; LVEDD: left ventricular end-diastolic dimension; LVESD: left ventricular end-systolic dimension; LVMI: left ventricular mass index.

* P<0.05 compared with group 1;

# P<0.05 compared with group 2;

+ P<0.05 compared with group 3.

The positive and negative predictive values, sensitivity, specificity, and accuracy of ECG-determined LVH in prediction of echocardiographic LVH were 67.6%, 61.3%, 38.4%, 84.1%, and 63.0% respectively. In addition, both ECG-determined LVH and echocardiographic LVH were significantly correlated with Ea and LVDD (all p<0.001).

Table 3 displays the univariate and multivariate linear regression analysis for Ea. Old age, history of diabetes, hypertension, and coronary artery disease, wide pulse pressure, increased heart rate, increased baPWV, ECG-determined LVH, high fasting glucose, ACEI use, ARB use, diuretic use, and nitrate use were significantly associated with low Ea in the univariate analysis. After multivariate analysis, old age, history of coronary artery disease, increased heart rate, diuretic use, increased baPWV, and ECG-determined LVH were independently associated with low Ea.

**Table 3:** Determinants of Ea in study patients.

Parameter		Univariate		Multivariate	
	R	Unstandardized coefficient β (95% CI)	P	Unstandardized coefficient β (95% CI)	P
Age (yr)	−0.494	−0.13 (−0.16, −0.11)	<0.001	−0.09 (−0.13, −0.05)	<0.001
Gender (F/M)	–	0.22 (−0.68, 1.12)	0.635	–	–
DM	–	−1.92 (−2.91, −0.92)	<0.001	–	–
HTN	–	−2.14 (−3.02, −1.26)	<0.001	–	–
CAD	–	−1.59 (−2.78, −0.41)	0.009	−1.03 (−2.03, −0.04)	0.043
PP (mmHg)	−0.241	−0.06 (−0.09, −0.03)	<0.001	–	–
HR (beat/min)	−0.167	−0.05 (−0.08, −0.01)	0.006	−0.05 (−0.08, −0.02)	0.003
baPWV (10cm/s)	−0.478	−0.04 (−0.05, −0.03)	<0.001	−0.02 (−0.04, −0.01)	<0.001
ECG-determined LVH	–	−2.30 (−3.28, −1.32)	<0.001	−1.77 (−2.66, −0.88)	<0.001
Laboratory parameters					
Fasting glucose (mg/dL)	−0.145	−0.01 (−0.02, −0.001)	0.035	–	–
Triglyceride (mg/dL)	−0.022	−0.001 (−0.005, −0.04)	0.751	–	–
Total cholesterol (mg/dL)	0.075	0.005 (−0.004, 0.01)	0.268	–	–
Hematocrit (%)	0.088	0.06 (−0.03, 0.14)	0.184	–	–
Anti-HTN medications					
ACEI use	–	−1.41 (−2.56, −0.26)	0.016	–	–
ARB use	–	−1.92 (−2.85, −0.98)	<0.001	–	–
β-blocker use	–	−0.54 (−1.45, 0.36)	0.239	–	–
CCB use	–	−1.03 (−2.07, 0.01)	0.052	–	–
Diuretics use	–	−2.46 (−3.58, −1.34)	<0.001	−1.69 (−2.67, −0.71)	0.001
Nitrate use	–	−1.81 (−2.73, −0.89)	<0.001	–	–

β: unstandardized coefficient; CI: confidence interval; ECG: electrocardiography; LVH: left ventricular hypertrophy; r: Pearson correlation coefficient. The other abbreviations are the same as in Tables 1 and 2.

Table 4 displays the univariate and multivariate logistic regression analysis for LVDD. Old age, history of diabetes and hypertension, wide pulse pressure, increased heart rate, increased baPWV, ECG-determined LVH, high fasting glucose, low hematocrit, ACEI use, ARB use, CCB use, diuretic use, and nitrate use were significantly correlated with LVDD in the univariate analysis. After multivariate analysis, old age, increased baPWV, and ECG-determined LVH were significantly correlated with LVDD.

**Table 4:** Determinants of LVDD in study patients.

Parameter	Univariate		Multivariate	
	OR (95% CI)	P	OR (95% CI)	P
Age (yr)	1.063 (1.04, 1.09)	<0.001	1.04 (1.01, 1.08)	0.028
Gender (F/M)	0.71 (0.44, 1.16)	0.172	–	–
DM	3.39 (1.79, 6.39)	<0.001	–	–
HTN	2.89 (1.74, 4.80)	<0.001	–	–
CAD	1.15 (0.60, 2.24)	0.67	–	–
PP (mmHg)	1.03 (1.01, 1.05)	0.002	–	–
HR (beat/min)	1.04 (1.02, 1.07)	<0.001	–	–
baPWV (10cm/s)	1.03 (1.02, 1.04)	<0.001	1.02 (1.01, 1.04)	0.011
ECG-determined LVH	3.48 (1.85, 6.56)	<0.001	3.53 (1.30, 9.55)	0.013
Laboratory parameters				
Fasting glucose (mg/dL)	1.01 (1.00, 1.02)	0.029	–	–
Triglyceride (mg/dL)	1.00 (0.99, 1.00)	0.766	–	–
Total cholesterol (mg/dL)	1.00 (0.99, 1.00)	0.432	–	–
Hematocrit (%)	0.95 (0.91, 1.00)	0.050	–	–
Anti-HTN medications				
ACEI use	2.50 (1.24, 5.05)	0.011	–	–
ARB use	2.66 (1.51, 4.67)	0.001	–	–
β-blocker use	0.93 (0.57, 1.52)	0.777	–	–
CCB use	2.17 (1.18, 3.99)	0.013	–	–
Diuretics use	2.46 (1.22, 4.98)	0.012	–	–
Nitrate use	1.89 (1.10, 3.23)	0.021	–	–

CI: confidence interval; ECG: electrocardiography; LVH: left ventricular hypertrophy; OR: Odds ratio. The other abbreviations are the same as in Tables 1 and 2.

## Discussion

There were three major findings in this study. First, Ea was gradually decreased from group 1 to group 4. Second, the group with higher baPWV and ECG-determined LVH by Sokolow-Lyon criterion had the highest prevalence of LVDD among the four study groups. Third, both baPWV and ECG-determined LVH by Sokolow-Lyon criterion were independently associated with Ea and LVDD.

It is important to clarify the characteristics of the four groups in our study. Compared with patients in group 3, patients in group 2 were younger and composed of fewer patients of hypertension, but LV systolic function was more decreased. The characteristics of these four groups could generally be defined as follows: patients in group 1 had relatively normal cardiovascular function, patients in group 2 had relative systolic dysfunction, patients in group 3 had relative vascular dysfunction, and patients in group 4 had relative cardiovascular dysfunction. Cardiovascular dysfunction progresses with arterial–cardiac interactions, but the progression of dysfunction differs in speed between the heart and the vessels. In some patients, cardiac dysfunction progresses first (group 2), in another patients, vascular dysfunction progresses predominantly (group 3), but in other patients, cardiovascular dysfunction may concurrently occur (group 4). In patients with combined heart and vascular disease, the baPWV may be high due to atherosclerotic vessels, but in patients with relatively pure heart disease such as idiopathic-dilated cardiomyopathy, the baPWV may be low because of reducing LV ejection velocity. Although patients in group 2 with relative systolic dysfunction had a low baPWV, they had a comparable prevalence of pseudonormal/restrictive mitral inflow pattern with patients in groups 3 and 4. Furthermore, when compared to patients in group 3, patients in group 4 had a higher prevalence of LVDD. Therefore, the presence or absence of ECG-determined LVH may be an important and useful data when interpreting the relationship between baPWV and LV diastolic function.

Increased PWV is an independent predictor of cardiovascular morbidity and mortality in the general population, hypertensive patients, the elderly, and patients with end-stage renal disease [24]–[26]. LVH is also associated with cardiovascular morbidity and mortality [27]–[29]. Furthermore, both PWV and LVH are reported to be associated with LVDD in the literature [3]–[7], [11]–[14]. Although the physiology of diastolic function is complex, the intrinsic LV abnormalities contributing to LVDD are as follows: (1) impaired LV relaxation, (2) increased LV asynchrony, and (3) the complex of LVH. LVH increases the ratio of myocardial mass to volume and the degree of hypertrophy is the main determinant of chamber stiffness. LVH often leads to poor LV compliance and a vicious cycle of greater LV filling pressures and hypertrophy. Ervin R. Fox et al. investigated the LV geometric patterns in a population-based African American cohort and found that concentric LVH was strongly associated with LVDD [14]. Hence, in this study, when comparing group 1 with group 2 and group 3 with group 4, patients with ECG-determined LVH (groups 2 and 4) had a higher prevalence of pseudonormal/restrictive mitral inflow pattern.

Aging has been reported to be associated with increased mean LV wall thickness, chamber diameter, mass, concentric remodeling, and a decline in LV diastolic function [30]. Samdarshi TE et al. showed that increased heart rate was a significant predictor of LVDD [31]. Rapid heart rate can cause increase in myocardial oxygen demand and decrease in coronary perfusion time, which promote ischemic LVDD even in the absence of coronary artery disease. In the present study, we consistently found old age and increased heart rate were the significant determinants of low Ea. In addition, diuretic use was also a significant determinant of Ea in this study. Diuretics were frequently used in patients with heart failure and fluid overload, which might partially explain the association between use of diuretics and Ea in this study.

About half of the patients with heart failure have preserved LV systolic function. Although heart failure with preserved ejection fraction may be uneasily recognized, it is associated with marked increases in morbidity and all-cause mortality [32]–[35]. Hence, identifying patients with LVDD is important. Although echocardiography has been a tool of choice for the assessment of LV diastolic function, it is still relatively expensive and operator-dependent. However, both ECG and ABI-form devices are relatively inexpensive and can be easily examined and interpreted without skilled operators. Hence, assessment of arterial stiffness by baPWV and LVH by ECG may be useful in identifying high-risk patients for LVDD if echocardiography and skilled operators are not available.

There are several limitations of this study. First, several criteria can be used to diagnose LVH via ECG [23], [36], but only the Sokolow-Lyon criterion was used in this study. Although the Sokolow-Lyon criterion is widely used by clinicians as it can be easily measured without complex calculations, its sensitivity in prediction of echocardiographic LVH may be low. Second, because our study was a cross-sectional one, we could only confirm the significant association of baPWV and ECG-determined LVH with Ea and LVDD. We could not elucidate the true cause-effect relationship among these. Third, many confounding factors might not be equally distributed among the groups being compared and this unequal distribution might lead to bias and possible misinterpretation. We minimized the possible confounding factors by statistically adjusting for them in multivariate analysis and still found that both baPWV and ECG-determined LVH were independently associated with Ea and LVDD. Forth, the majority of our patients were treated chronically with antihypertensive medications. For ethical reasons, we did not withdraw these medications. Hence, we could not exclude the influence of antihypertensive agents on our findings. Fifth, as no large-scale studies have documented the reliable value of baPWV in prediction of increased arterial stiffness, we used median value of baPWV to classify our study patients. In addition, LVDD was defined on the basis of noninvasive data rather than by invasive measurements. The results might be somewhat different if invasive data were used. Finally, since the subjects of this study were already being evaluated for heart disease, it was susceptible to selection bias and making findings potentially less generalized.

In conclusion, the present study demonstrated that Ea was gradually decreased from group 1 to group 4 and the group with higher baPWV and ECG-determined LVH by Sokolow-Lyon criterion had the highest prevalence of LVDD among the four study groups. In addition, both baPWV and ECG-determined LVH as measured by the Sokolow-Lyon criterion were independently associated with Ea and LVDD. Hence, assessment of arterial stiffness by baPWV and LVH by ECG may be useful in identifying high-risk patients for LVDD.
